# Phylogenomics Reveals that *Asaia* Symbionts from Insects Underwent Convergent Genome Reduction, Preserving an Insecticide-Degrading Gene

**DOI:** 10.1128/mBio.00106-21

**Published:** 2021-03-30

**Authors:** Francesco Comandatore, Claudia Damiani, Alessia Cappelli, Paulo Eduardo Martins Ribolla, Giuliano Gasperi, Francesco Gradoni, Gioia Capelli, Aurora Piazza, Fabrizio Montarsi, Maria Vittoria Mancini, Paolo Rossi, Irene Ricci, Claudio Bandi, Guido Favia

**Affiliations:** a Pediatric CRC Romeo ed Enrica Invernizzi, Department of Biosciences, Università di Milano, Milan, Italy; b School of Biosciences & Veterinary Medicine, University of Camerino, Camerino, MC, Italy; c CIRM Italian Malaria Network, Unit of Camerino, MC, Italy; d Biotechnology Institute (IBTEC), Sao Paulo State University (UNESP), Sao Paulo, Brazil; e Biosciences Institute at Botucatu (IBB), Sao Paulo State University (UNESP), Sao Paulo, Brazil; f Department of Biology and Biotechnology, University of Pavia, Pavia, Italy; g Istituto Zooprofilattico Sperimentale delle Venezie, Legnaro (Padua), Italy; h MRC-University of Glasgow Centre for Virus Research, Glasgow, United Kingdom; i Pediatric CRC Romeo ed Enrica Invernizzi, DIBIC, Università di Milano, Milan, Italy; GSK Vaccines

**Keywords:** *Asaia*, genome reduction, pyrethroid hydrolase

## Abstract

The mosquito microbiota is composed of several lineages of microorganisms whose ecological roles and evolutionary histories have yet to be investigated in depth. Among these microorganisms, *Asaia* bacteria play a prominent role, given their abundance in the gut, reproductive organs, and salivary glands of different mosquito species, while their presence has also been reported in several other insects. Notably, *Asaia* has great potential as a tool for the control of mosquito-borne diseases. Here, we present a wide phylogenomic analysis of *Asaia* strains isolated from different species of mosquito vectors and from different populations of the Mediterranean fruit fly (medfly), *Ceratitis capitata*, an insect pest of worldwide economic importance. We show that phylogenetically distant lineages of *Asaia* experienced independent genome reductions, despite following a common pattern, characterized by the early loss of genes involved in genome stability. This result highlights the role of specific metabolic pathways in the symbiotic relationship between *Asaia* and the insect host. Finally, we discovered that all but one of the *Asaia* strains included in the study possess the pyrethroid hydrolase gene. Phylogenetic analysis revealed that this gene is ancestral in *Asaia*, strongly suggesting that it played a role in the establishment of the symbiotic association between these bacteria and the mosquito hosts. We propose that this gene from the symbiont contributed to initial pyrethroid resistance in insects harboring *Asaia*, also considering the widespread production of pyrethrins by several plants.

## Abstract (precis)

We have studied genome reduction within several strains of the insect symbiont *Asaia* isolated from different species/strains of mosquito and medfly. Phylogenetically distant strains of *Asaia*, despite following a common pattern involving the loss of genes related to genome stability, have undergone independent genome reductions, highlighting the peculiar role of specific metabolic pathways in the symbiotic relationship between *Asaia* and its host.

## INTRODUCTION

Symbiosis shaped the evolution of animals and plants, providing new adaptive traits to multicellular organisms and increasing their ability to create and colonize novel ecological niches (1). On the other hand, microbial symbionts have also been subjected to drastic functional and metabolic modifications, associated with a reduction of their genome sizes (2). The phenomenon of bacterial genome reduction has been shown to correlate with genome-wide loss of genes. This is particularly well described for obligate bacterial endosymbionts of insects, such as Blattabacterium cuenoti, Buchnera aphidicola, and *Carsonella ruddii* (3, 4).

We recently provided the first evidence of genome reduction in *Asaia* (5), a major component of the gut microbiota in several insects, including relevant human disease vectors (6). Indeed, *Asaia* has been detected in *Anopheles*, *Aedes*, and *Culex* mosquito species, which are known to transmit several pathogens to humans, including the etiological agents of malaria, dengue, yellow fever, zika, chikungunya, and some filarial diseases (6–10). Notably, *Asaia*, apart from its well-established role as a main component of the insect microbiota, can also be regarded as an environmental bacterium generally associated with sugar-containing water pools, such as those in flowers and leaf axils in several plants (11).

In our previous studies, we showed that *Asaia* influences both the development and the longevity of Anopheles stephensi mosquitoes (12, 13). Moreover, experimental evidence indicates that *Asaia* affects immune-related gene expression in mosquitoes (13–15). Overall, the collective evidence suggests that *Asaia* plays a crucial role in mosquito development, physiology, and survival.

Interestingly, *Asaia* has also been detected in insect pests and vectors of agricultural relevance, such as Scaphoideus titanus and the white-backed planthopper, *Sogatella furcifera* (6, 16). In this context, we have recently detected *Asaia* in different strains of *Ceratitis capitata* (C. Damiani et al., unpublished data), a major pest threat to agriculture in several geographical regions worldwide (17).

Our previous study showed that *Asaia* from Anopheles darlingi, a South American malaria vector (5), underwent a significant reduction both in genome size and in gene content. Consequently, *Asaia* was proposed as a novel model to investigate genome reduction; we approach this phenomenon within a single bacterial taxon that includes both environmental and insect-associated strains or species.

Here, we present a comprehensive comparative genomics analysis of different *Asaia* strains isolated from different sources, including several mosquito species, different populations of *C. capitata*, and environmental samples. Our results revealed substantial changes in genomic content across different *Asaia* lineages, showing independent genome reduction processes that occurred through a common pattern. Furthermore, we found that all but one of the *Asaia* strains examined harbor the pyrethroid hydrolase (PH) gene, which is likely to confer resistance to pyrethroids, insecticides produced by plants and commonly used in pest treatments (18).

## RESULTS AND DISCUSSION

A total of 17 novel genomes were obtained from *Asaia* strains from mosquitoes and Ceratitis capitata; these were compared with the 13 *Asaia* genomes already available in the database, obtained from stains isolated from mosquitoes and from environmental samples (see Table S1 in the supplemental material).

10.1128/mBio.00106-21.1 TABLE S1 *Asaia* strain information. Download Table S1, PDF file, 0.07 MB. Copyright © 2021 Comandatore et al. 2021 Comandatore et al. https://creativecommons.org/licenses/by/4.0/ This content is distributed under the terms of the Creative Commons Attribution 4.0 International license.

**FIG 1:**
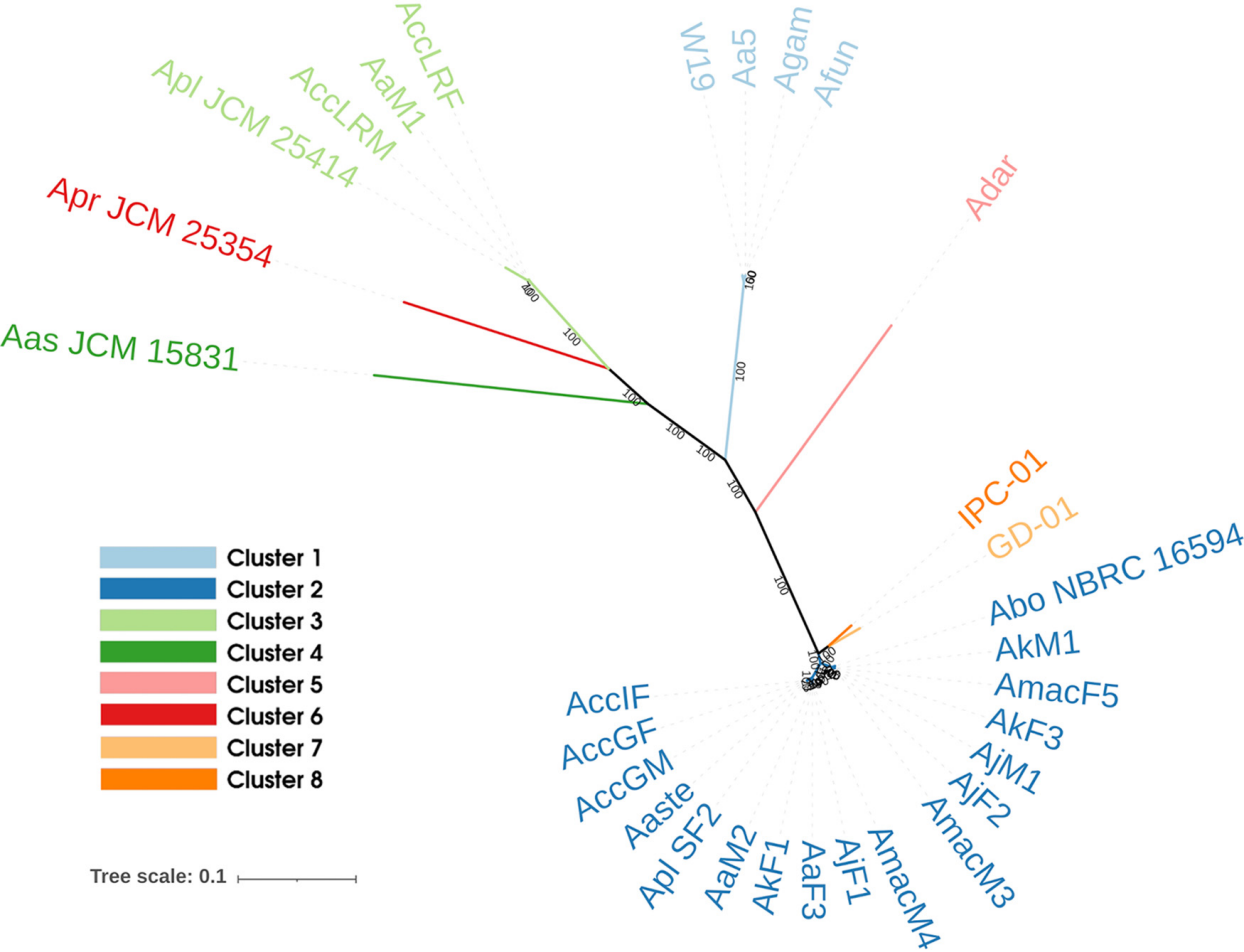
Maximum-likelihood phylogenetic tree obtained using the 612 core genes among the 30 *Asaia* species strains included in the study and the *Gluconobacter morbifer* strain G707 as the outgroup. Tree leaves and the relative monophyletic clade colors represent the clusters identified on the basis of average nucleotide identity (ANI), with a cutoff of 85%. Bootstrap support values are reported on the branches of the tree.

Orthologous analysis allowed the identification of 612 core genes for an amino acid concatenate, sized 183,373 bp. The obtained maximum-likelihood (ML) phylogenetic tree is shown in Fig. 1. Average nucleotide identity (ANI) analysis clustered *Asaia* strains into six subgroups, corresponding to the previously described *Asaia* species (Fig. 1). An integrative analysis has been performed with a larger number of outgroups, confirming the divergent clustering of *Asaia* while retaining the six subgroups (Fig. S1).

10.1128/mBio.00106-21.4 FIG S1 Maximum-likelihood phylogenetic tree, including the study strains of the genus *Asaia* and 12 strains of four genera belonging to the *Acetobacteraceae* family. The phylogenetic analysis was performed on a concatenate of 356 core genes. These core genes were selected as follows: present in single copy in all the genomes and without frameshifts or stop codons in the alignment. Bootstrap supporting values are reported on the tree branches. Download FIG S1, PDF file, 0.1 MB. Copyright © 2021 Comandatore et al. 2021 Comandatore et al. https://creativecommons.org/licenses/by/4.0/ This content is distributed under the terms of the Creative Commons Attribution 4.0 International license.

It is worth nothing that the current taxonomic classification of the *Asaia* strains is debated; indeed most of the sequenced strains are not officially assigned to specific *Asaia* species. The ANI-based analysis with a 95% threshold is often used to describe bacterial species (19), suggesting that the “clusters” that we found may correspond to *Asaia* species. Coherently, we retain the term “cluster.”

**FIG 2:**
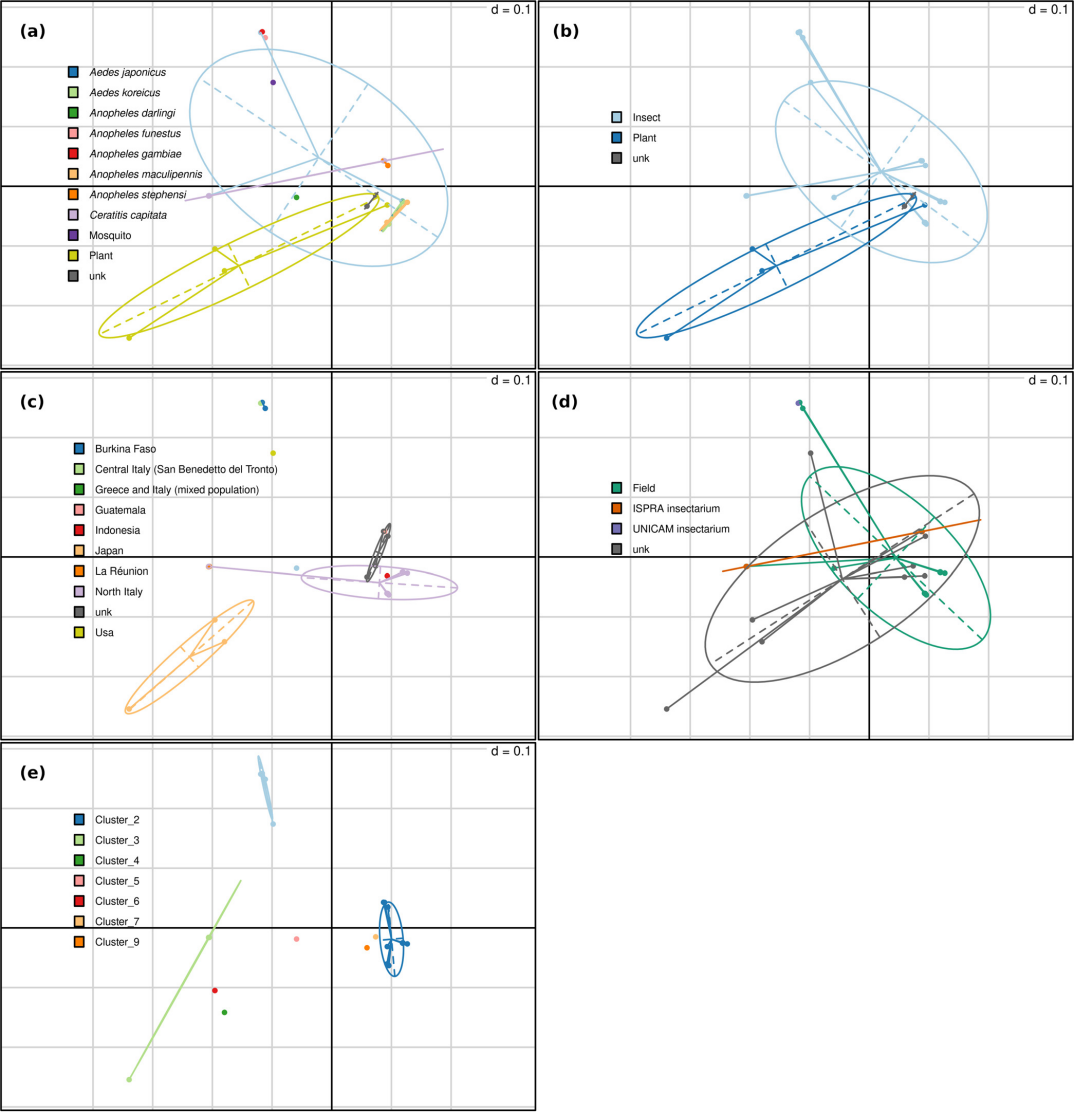
Bidimensional principal-coordinate analysis plots generated on the basis of gene presence/absence (explained variance, 24.54% on the *x* axis and 16.10% on the *y* axis). Each dot of the plots represents one of the 30 *Asaia* species strains included in the study. (a) Each color corresponds to a single host species. (b) *Asaia* hosts are grouped in the categories insects, plants, and unknown. (c) *Asaia* hosts are grouped on the basis of the sampling geographic location. (d) *Asaia* hosts are grouped on the basis of the sample origin. (e) *Asaia* hosts are grouped on the basis of clusters identified using ANI, with a cutoff of 95%.

The variance underlying the specific clustering of the different *Asaia* strains reflects the existence of differential gene compositions, thus suggesting an evolution that occurred through independent genomic reduction/variation pathways. A principal-coordinate analysis (PCoA) of gene presence/absence across the different *Asaia* strains analyzed clearly indicates that these differences are not solely attributable to ecological adaptations (Fig. 2); they should also be interpreted as a consequence of the speciation process.

**FIG 3:**
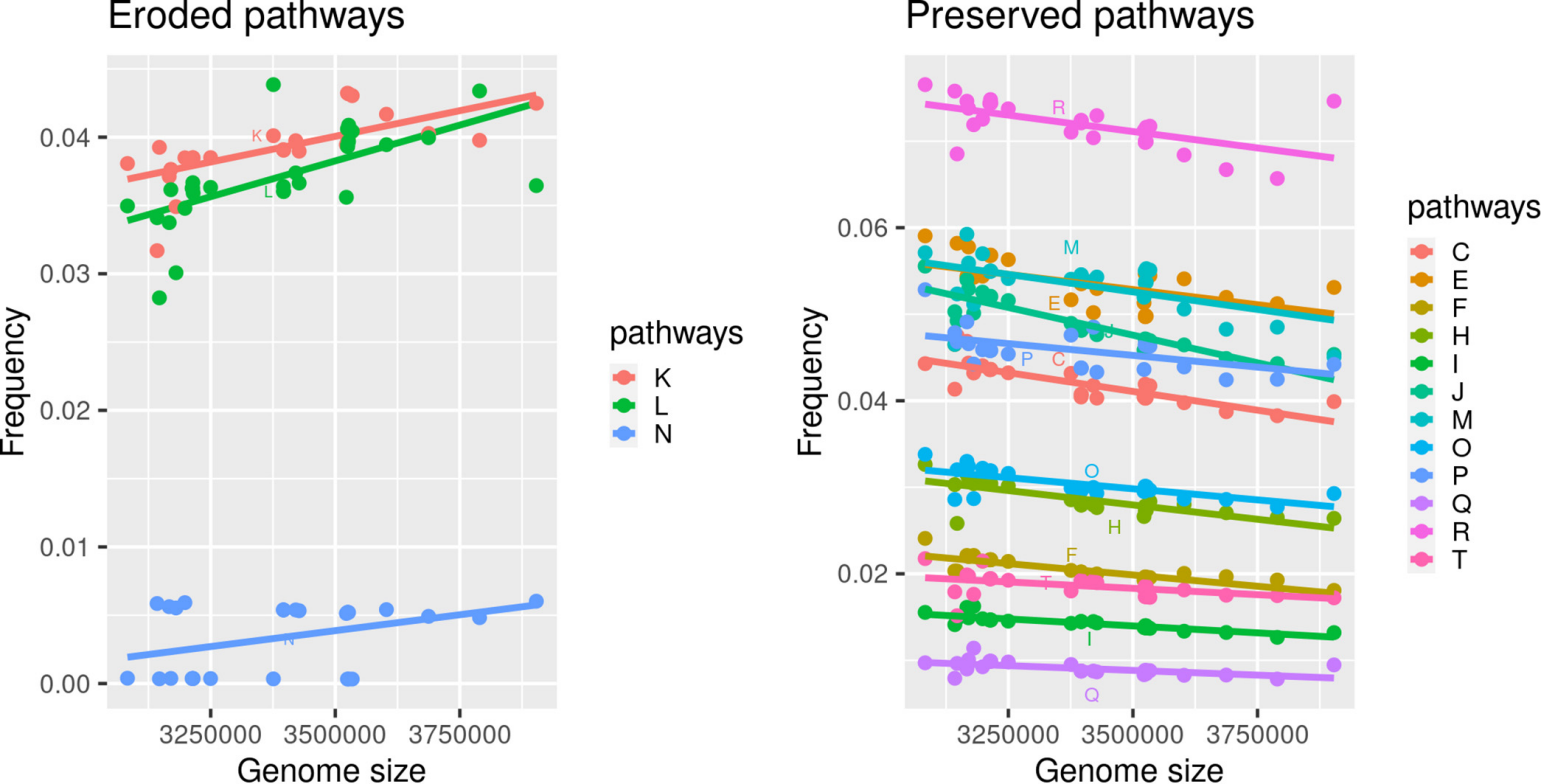
Pathway erosion. For each of the 30 *Asaia* species strains included in the study, the proportion of genes belonging to each cluster of orthologous groups (COG) was computed as the ratio between the number of genes belonging to a specific pathway and the total number of genes. The scatterplots of COG pathways for which we found a significant linear correlation (Spearman test, *P* value < 0.05) between pathway proportion and genome size are shown. In the plot on the left, the COG pathways showing a positive correlation are reported, and in the plot on the right, those showing a negative correlation are reported. A positive correlation suggests that a pathway has been subjected to erosion during genome reduction; conversely, a negative correlation suggests its preservation.

Even if the presented evidence suggests an independent evolution of the different *Asaia* strains, we found that genomic erosion tends to preserve some pathways, while others erode more easily in all strains (Fig. 3). In particular, only the “replication, recombination, and repair” (L), “transcription” (K), and “cell motility” (N) cluster of orthologous groups (COG) pathways showed a clear erosion trend (Fig. 3, left), while several others were preserved (Fig. 3, right; Table S2). Notably, most of the preserved pathways are involved in essential metabolic functions of the bacteria (e.g., transport and metabolism of carbohydrates, amino acids, nucleotides, and coenzymes, as well as membrane biogenesis and energy production). This clearly suggests that the erosion process tends to preserve genomic stability *tout-court* pathways rather than being subjected to functional/ecological adaptation processes, at least in the early stages of reduction.

10.1128/mBio.00106-21.2 TABLE S2 Pathway linear regression analysis information. Download Table S2, PDF file, 0.04 MB. Copyright © 2021 Comandatore et al. 2021 Comandatore et al. https://creativecommons.org/licenses/by/4.0/ This content is distributed under the terms of the Creative Commons Attribution 4.0 International license.

**FIG 4:**
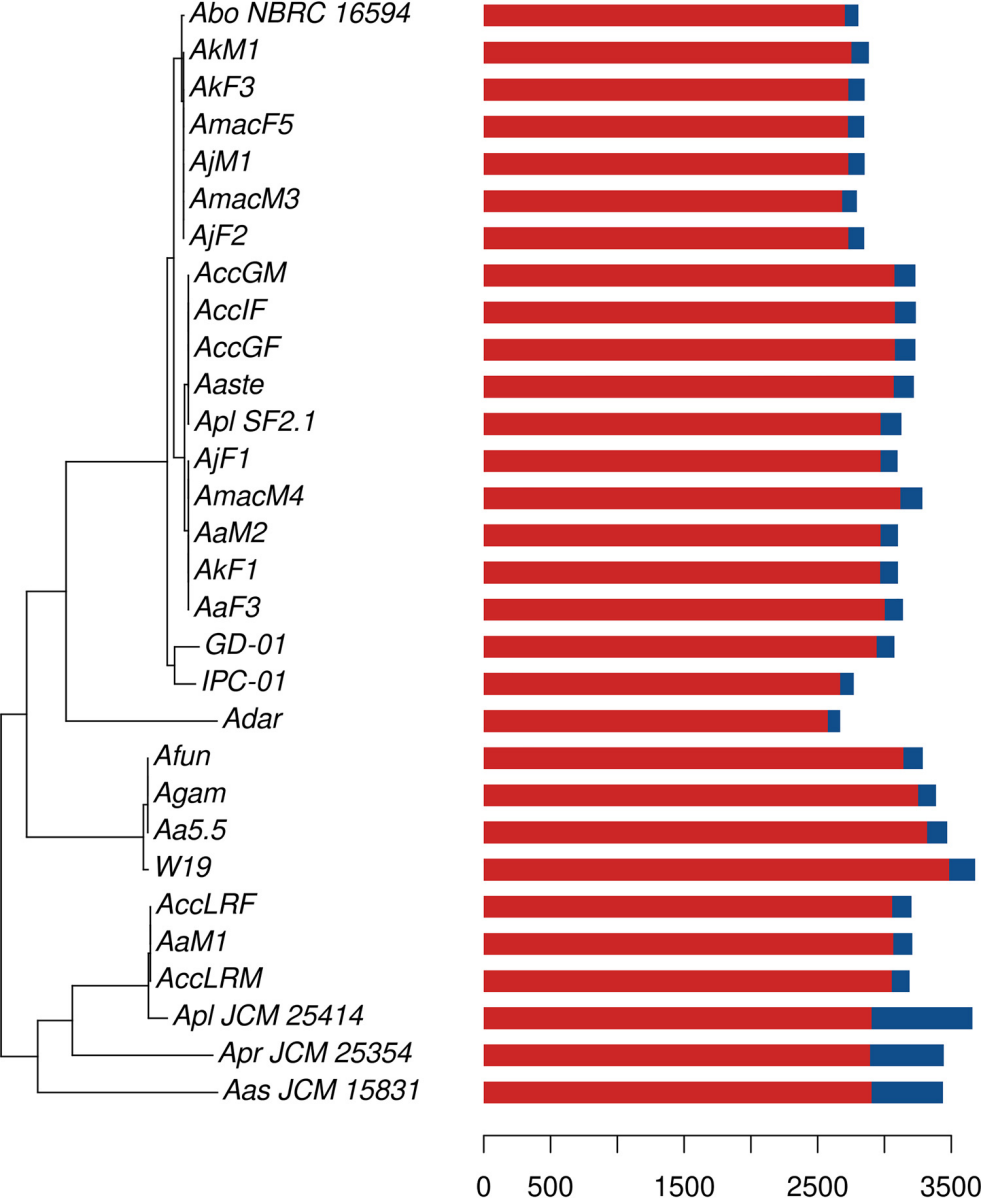
Eroded and entire genes among *Asaia* species genomes. Maximum-likelihood phylogenetic tree obtained using the 612 core genes among the 30 *Asaia* species strains included in the study and *Gluconobacter morbifer* strain G707 (outgroup). The tree has been rooted on the outgroup strain, which was then removed. The bar plot reports the total number of pseudogenes (in blue) and nonpseudogenes (in red).

Interestingly, the erosion patterns of the L, K, and N pathways are conserved during the independent genome reduction processes occurring across the different *Asaia* strains (Fig. 4). These results show that genome erosion pathways follow a conserved stream, likely driven by metabolic constraints. It is also interesting to note that all the *Asaia* strains harbor the *recA* gene, usually lost in highly reduced genomes (20, 21). Furthermore, three plant-derived strains show a slightly different pattern of erosion with regard to the two pathways with the highest percentages of eroded genes (Fig. 3). This suggests that, despite the two pathways being associated with cellular/genomic stability, the environment still plays an important role in the definition of the genomic erosion process. This different erosion pattern may be a consequence of a strategy to evade the Muller’s ratchet phenomenon (22). As proposed by Naito and Pawlowska (23), the preservation of genes involved in genome stability (e.g., recombination genes) can reduce the accumulation of disadvantageous mutations due to the bottlenecks associated with mother-to-offspring bacterial transmission (22). We can speculate that *Asaia* strains associated with insects experience bottlenecks more frequently than those living in the environment.

**FIG 5:**
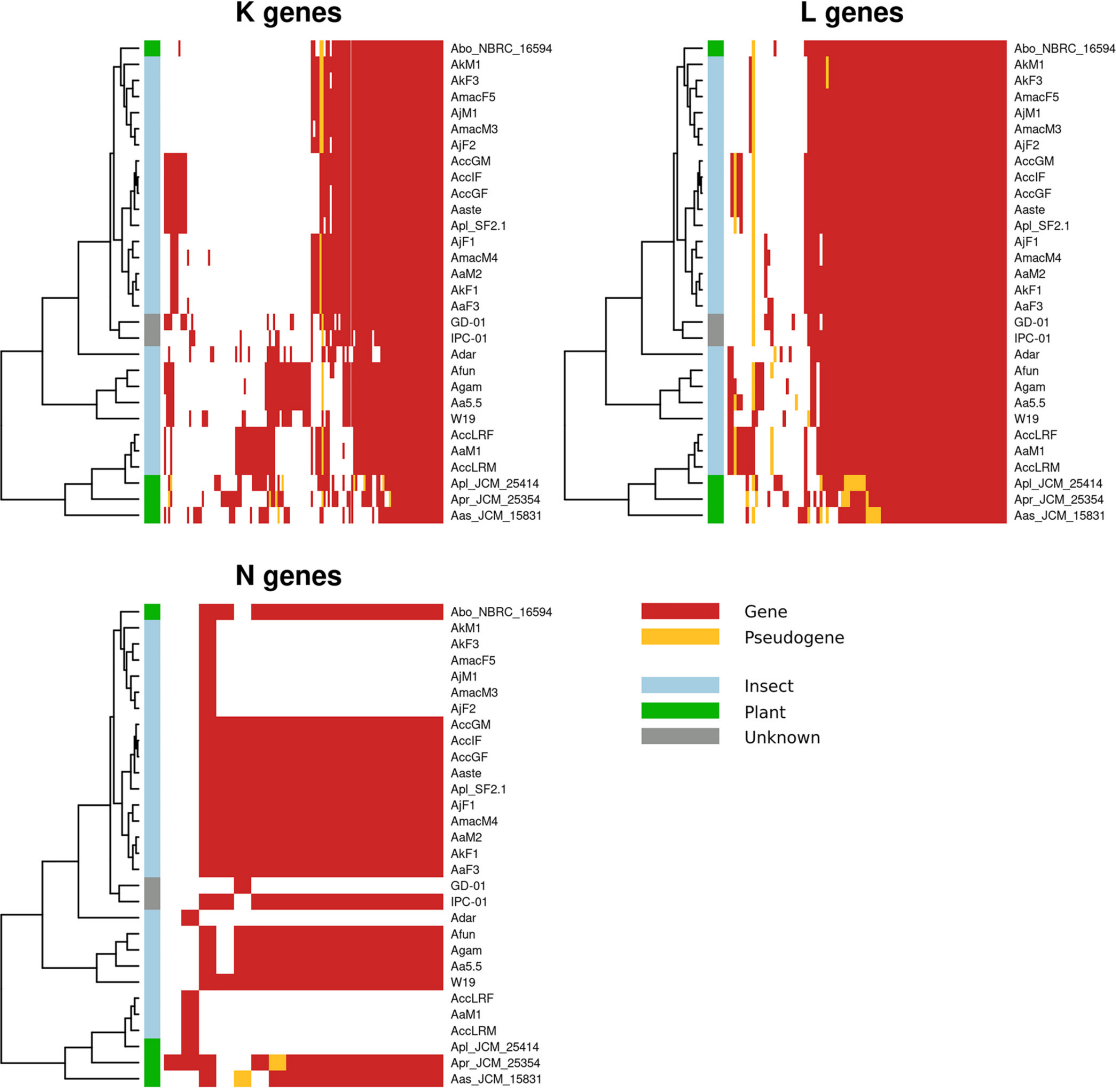
Eroded COG pathways. A heatmap of insect, plant, and unknown gene presence/absence in the eroded COG pathways K (transcription), L (replication, recombination, and repair), and N (cell motility) is reported. On the left of each heatmap plot, we show the maximum-likelihood phylogenetic tree of the 30 *Asaia* species strains included in the study, in the middle, we show a colored bar of the strain isolation sources (see the color key), and on the right, the gene/pseudogene presence/absence information is reported (see the color key; absent genes are white). In each heatmap, the genes are clustered as obtained using the hclust R function.

Moreover, a heatmap that takes into account only the presence of pseudogenes clearly demonstrates that some genes are eroded in many organisms (Fig. 4 and 5). It also shows that each *Asaia* strain isolated from plants, phylogenetically distant from those isolated from insects (Fig. 1), belongs to a different cluster denoting differentiated reduction patterns. These results suggest a parallel and convergent reduction process of *Asaia* strains from insects.

**FIG 6:**
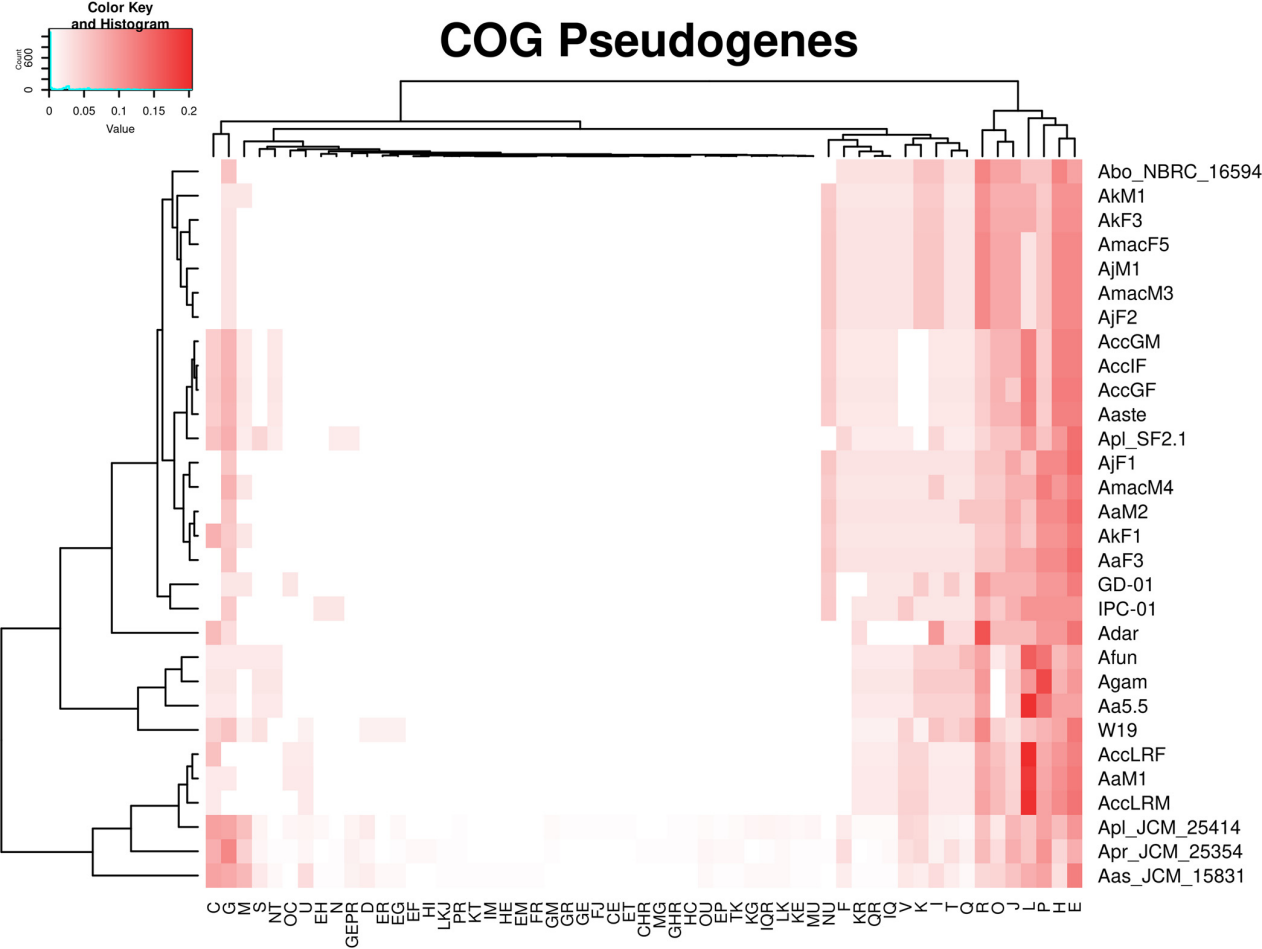
Proportions of pseudogenes among COG pathways. On the left, the maximum-likelihood phylogenetic tree obtained using core genes was rooted on the outgroup (and then removed). On the right, colors represent the proportions of pseudogenes present in each COG pathway in each strain. For each strain, the proportion is computed as the ratio between the number of pseudogenes present in a COG pathway and the total number of pseudogenes. (COG pathways explanation: J, translation, ribosomal structure and biogenesis; A, RNA processing and modification; K, transcription; L, replication, recombination and repair; B, chromatin structure and dynamics; D, cell cycle control, cell division, chromosome partitioning; Y, nuclear structure; V, defense mechanisms; T, signal transduction mechanisms; M, cell wall/membrane/envelope biogenesis; N, cell motility; Z, cytoskeleton; W, extracellular structures; U, intracellular trafficking, secretion, and vesicular transport; O, posttranslational modification, protein turnover, chaperones; C, energy production and conversion; G, carbohydrate transport and metabolism; E, amino acid transport and metabolism; F, nucleotide transport and metabolism; H, coenzyme transport and metabolism; I, lipid transport and metabolism; P, inorganic ion transport and metabolism; Q, secondary metabolites biosynthesis, transport and catabolism; R, general function prediction only; S, function unknown. When more letters are reported, the gene is classified in more than one COG pathway.)

We have produced an additional heatmap where the frequencies of pseudogenes belonging to each COG cluster are reported for each organism (Fig. 6). This analysis reveals a substantial convergence of reductions of pathways, excepting for the three *Asaia* isolates from plants, which tend to reduce some pathways more than others. Interestingly, the frequency of pseudogenes of the transcription pathway (K) is not very high; it occurs as well in the cell motility pathway (N). The replication, recombination, and repair (L) pathway, on the other hand, is one of the richest in pseudogenes. This may be due to the fact that the erosion of the K and N pathways is recent; therefore, we observe only a limited number of pseudogenes. On the other hand, the erosion of the L pathway seems to be a more gradual process and therefore to be considered ongoing. All of this seems in accordance with the hypothesis of escape from Muller’s ratchet: in order not to accumulate deleterious mutations, the pseudogenes preserve the path of recombination while eroding the other two pathways much faster. Interestingly, while the erosion of the L pathway (recombination) is fairly well conserved among the different lineages (Fig. 6), this does not apply to the K pathway, which shows blocks of different genes conserved through the different lineages. This means that the reduction of the transcriptional apparatus can reach different stable “configurations.”

Thus, we focused on some genes and/or metabolic patterns on which we had focused in our previous studies because they are maintained across different *Asaia* strains (5, 13).

**FIG 7:**
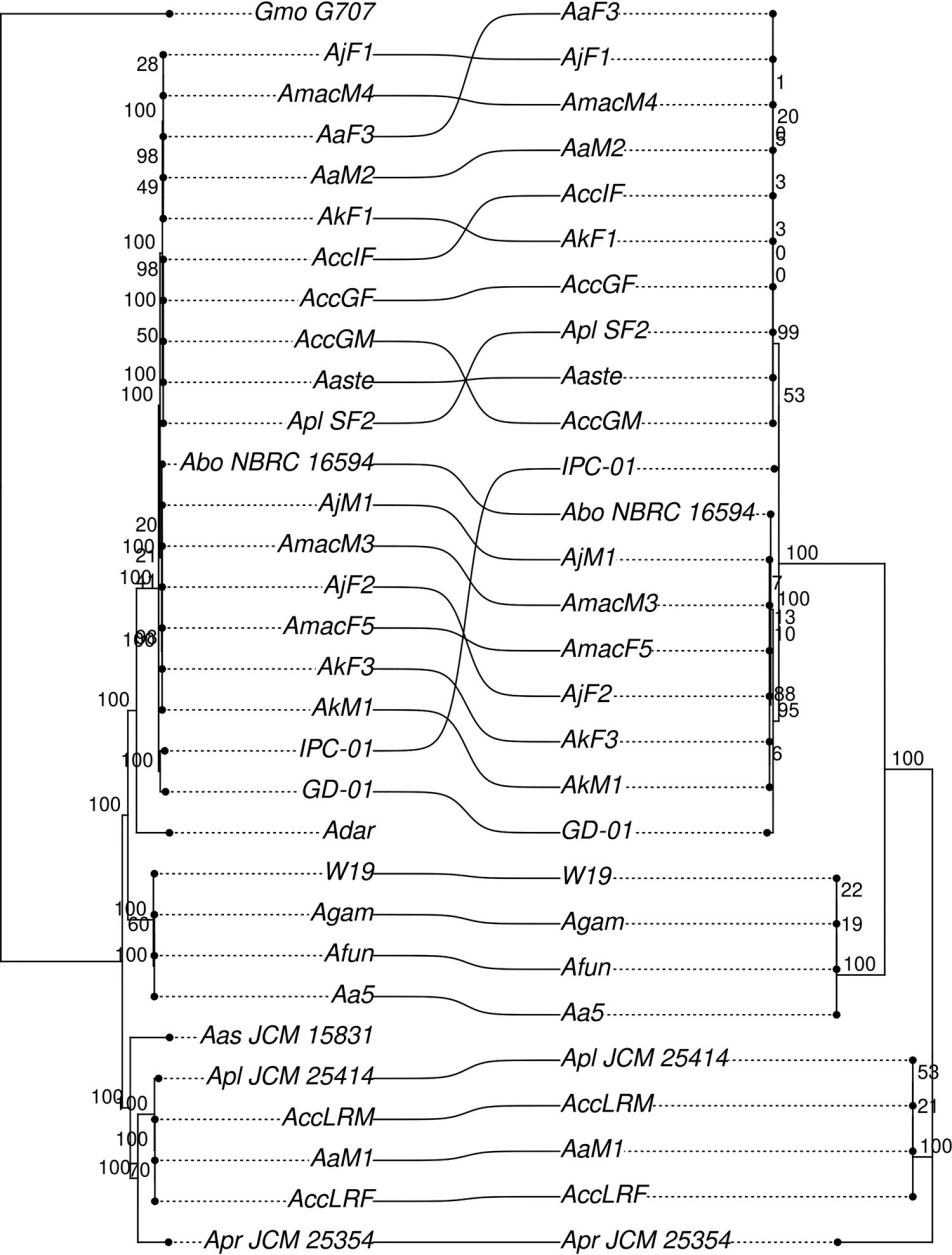
Comparison of an *Asaia* species tree and a pyrethroid hydrolase phylogenetic tree. On the left is the maximum-likelihood phylogenetic tree obtained using 612 core genes shared among the 30 *Asaia* species strains and the outgroup (*Gluconobacter morbifer* [*Gmo*] strain G707). On the right is the maximum-likelihood phylogenetic tree reconstructed on the basis of the pyrethroid hydrolase amino acid sequence. Bootstrap supports are reported for both trees. Strains shared among the two trees are connected by curved lines. The absence of the lines relative to Adar strain and the outgroup (*Gmo* G707) indicates the absence of the pyrethroid hydrolase gene in their genomes.

In the first instance, we turned our attention to the pyrethroid hydrolase (PH) gene, which regulates pyrethroid degradation and whose mosquito counterpart is known to play a role in pyrethroid resistance. We therefore hypothesized what role it may play in the biology of *Asaia*. The gene coding for PH was found to be present in all but one *Asaia* strains and not only in those isolated from insects. Interestingly, this gene is not present in *Asaia* strains isolated from *An. darlingi*, which have the smallest bacterial genomes among all those analyzed in this study. The consistency between the phylogenetic analysis of the gene and the phylogenomic analysis reveals that this gene is probably an ancestral character of the genus *Asaia* (Fig. 7). Accordingly, it is possible to hypothesize different roles for PH in the biology of the bacterium. In fact, given the recognized role of PH in the protection against pyrethroids (which are also present in nature, as they are produced by some plants), the retention of the PH gene may be for its own protection or to indirectly protect the insect host from pyrethroid toxicity.

Consequently, the *Asaia* strain isolated from *An. darlingi* missing the PH gene stands out as an ideal candidate to test this hypothesis, above all in light of a recent study aimed at evaluating the susceptibility of *An. darlingi* and Anopheles marajoara to pyrethroids used by the National Malaria Control Program in Brazil. This study showed that no resistance to cypermethrin, deltamethrin, or alpha-cypermethrin was recorded for *An. darlingi*, while diagnostic doses estimated for *An. marajoara* indicate that this species requires attention (24). It is important to remark that changes in *Anopheles* vector competence for malaria parasites have been linked to its insecticide resistance status. In fact, insecticide exposure is detrimental to *Plasmodium* in the midgut lumen, and it is more than likely that additional resistance mechanisms impact parasite development within the vector host (25). It is worth mentioning that there are already several examples of insect symbionts that contribute in whole or in part to the lower resistance of the host to the insecticide. In 2012, Kikuchi and collaborators demonstrated that the fenitrothion-degrading *Burkholderia* strains establish a specific and beneficial symbiosis with stinkbugs and confer a resistance to fenitrothion to the host insects (26). More recently, it was proven that *Hamiltonella defensa* can have an impact on host aphid susceptibility to some insecticides. In fact, compared with *Hamiltonella-*free aphid clones, *Hamiltonella*-infected aphid clones exhibit lower sensitivity to most of the tested insecticides at low concentrations (27). More generally, although the mechanisms of resistance to insecticides are diversified, detoxification from insecticides mediated by symbionts within insect pests and vectors is a trend that has now become clear and that is configured as an emerging problem in the control of these insects (28).

The second group of genes that has been considered is the one responsible for motility. Our previous study has shown that the genome of the *Asaia* strain isolated from *An. darlingi* had lost a large group of genes involved in the flagellar machinery (i.e., flagellum biosynthesis proteins, flagellar basal-body rod proteins, flagellar motor rotation proteins) (5). In particular, we identified both mobile and nonmobile strains of *Asaia* (Table S1). Moreover, it is worth mentioning that swimming motility has been implicated in gut colonization of several hosts, influencing also pathogen infection in mosquitoes (29).

Mobile strains of *Asaia* have larger genomes than immobile strains (Wilcoxon test, *P* value = 0.001). Nonetheless, the analysis showed that genes involved in twitching and in pilus and biofilm production are present in both mobile and immobile strains. These results are greatly supportive of the notion that the acquisition of environmental symbionts often necessitates active migration and colonization by the symbionts by means of motility and chemotaxis (30).

We had already proposed the insect symbiont *Asaia* as a novel model to study genome reduction dynamics within a single bacterial taxon evolving in a common biological niche. In contrast to what usually happens in comparison studies of genomic reduction involving genomes substantially different from each other in size (31), in the present study, differences in genomic size fluctuate in a 0.5-Mb range. Conserved patterns between independent lines in this data set suggest that genomic reduction may be an ongoing biological process within the *Asaia* genus, thus confirming this insect symbiont as a model for genome reduction studies.

This study pinpoints the importance of the environment in the definition of the genomic erosion process, selecting and preserving those genes involved in genome stability. On the other hand, our study highlights the role of specific genes in the host’s biology. In particular, the identification of the pyrethroid hydrolase gene in almost all the *Asaia* strains isolated, besides the one from the South American malaria vector *An. darlingi*, for which resistance to pyrethroids has never been reported, opens the way to a possible involvement of bacterial symbionts in determining the resistance to insecticides developed by insect vectors and insect pests. If confirmed, this involvement would require a revision of the control methods of insect vectors and pests currently in use.

## MATERIALS AND METHODS

### *Asaia* strain collection.

*Asaia* strains were obtained from different mosquito species and from three different *C. capitata* populations. *Asaia* strain details are listed and described in Table S1 in the supplemental material. Bacterial isolations were performed as reported in the work of Favia et al. (7). Briefly, the insect surface bodies were sterilized by washing them twice in phosphate-buffered saline (PBS) and 70% ethanol. After homogenization, *Asaia* was isolated from a pool of five insects by a preenrichment step in liquid atomic block element modifier (ABEM) medium (2% sorbitol, 0.5% peptone, 0.3% yeast extract, 100 μg/ml actidione, pH 3.5) and hence plated in agarized medium (2% glucose, 0.5% ethanol, 0.8% yeast extract, 0.7% carbonate calcium, 1.2% agar, pH 7). *Asaia* colonies were identified by their morphology and ability to produce carbonate dissolution haloes in agar plates. Identification was then confirmed by PCR using 1492Rev-27For oligonucleotides targeting the 16S rRNA gene and sequencing by Sanger methods (Eurofins, Germany). The *Asaia* motility test was conducted using the hanging drop procedure. Each bacterial strain was grown on GLY agar (yeast extract 1%, glycerol 2.5%, agar 2%, pH 5) for 48 h at 30°C. After a single colony was picked from each plate, bacteria were resuspended in a drop of 1× PBS and then observed under ×100 magnification optical microscopy. Cell motility was assessed by observing multiple areas of the same slide within three biological replicates (Table S1). The *Asaia* genome sequences of environmental strains were recovered from the PATRIC database (Table S3).

10.1128/mBio.00106-21.3 TABLE S3 Genome information and statistics. Download Table S3, PDF file, 0.05 MB. Copyright © 2021 Comandatore et al. 2021 Comandatore et al. https://creativecommons.org/licenses/by/4.0/ This content is distributed under the terms of the Creative Commons Attribution 4.0 International license.

### DNA extraction and genome sequencing.

The 17 *Asaia* species strains were subjected to whole DNA extraction and whole-genome sequencing (WGS). Genomic DNA was extracted using the QIAamp DNA minikit (Qiagen) by following the manufacturer’s instructions, and WGS was performed using the Illumina MiSeq platform with a 2 × 250 paired-end run after Nextera XT library preparation (Illumina Inc., San Diego, CA).

### Genome retrieving.

All the genome assemblies and genomic reads of *Asaia* species strains available in public databases on 1 March 2020 were retrieved, for a total of eight strain genome assemblies (retrieved from the NCBI and PATRIC) and five strain short-read data files (retrieved from Sequence Read Archive [SRA]). Furthermore, the genome assembly of *Gluconobacter morbifer* strain Gmo_G707, included as outgroup in the phylogenomic analyses, was retrieved from the NCBI. For more details, see Table S3.

### Genome assembly.

The paired-end reads obtained from the 17 *Asaia* strains and the five read sequences retrieved from the SRA were subjected to a quality check using the FastQC tool (32) and then assembled using SPAdes 3.13 (33).

### ANI-based genome clustering.

The pairwise average nucleotide identity (ANI) values among the 31 genomes included in the study were computed using the OrthoANI tool (34). Genomes were then clustered using a cutoff threshold of 95% (19).

### Genome annotation and orthologous analysis.

All the 31 genome assemblies included in the study were subjected to open reading frame (ORF) calling and annotation using PROKKA (35). Amino acid sequences obtained from PROKKA for all the genome assemblies were then used for orthologous analysis using OrthoMCL (36). The output of the orthologous analysis was then refined using the annotation information; two sequences were included in the same orthologous group if they belonged to the same OrthoMCL orthologous group and they had the same PROKKA annotation.

### Identification of pseudogenes.

For each refined orthologous group, the sequences were retrieved, aligned, and analyzed to identify pseudogenes. We classified as pseudogenes the sequences containing one (or more) internal stop codon and one (or more) frameshift or those sequences for which > 30% of the bases in the alignment were gaps. For more detail, for each group, the amino acid sequences were aligned using MUSCLE (37) and the obtained alignment was used to obtain the relative codon-based nucleotide alignment. The obtained nucleotide alignments were then analyzed using MACSE 2.03 (38) to identify internal stop codons and frameshifts. The percentage of gap positions for each sequence of each nucleotide alignment was obtained using a Perl script.

### Phylogenomic analysis.

Core genes were identified as pseudogene-free orthologous groups containing one gene sequence from each of the 31 genome assemblies included in the study. Amino acid alignments of core genes were retrieved, concatenated, and subjected to maximum-likelihood (ML) phylogenetic analysis. The ML phylogenetic analysis was conducted using RAxML 8 (39) with the LG+I+G+F model, selected using ProtTest3 (40).

### PCoA.

The output of orthologous analysis was converted in a gene presence/absence matrix, and principal-coordinate analysis (PCoA) was performed using R.

### Pathway erosion analysis.

To investigate relationship between pathway erosion and genome size, we annotated all the amino acid sequences of the 30 *Asaia* strain genomes by a BLASTP search against the clusters of orthologous groups (COGs) in the protein database. Then, for each genome, we computed the proportion of genes of each COG pathway as the ratio between the number of the genes belonging to each COG pathway and the total number of genes. Then, for each COG pathway, we computed the linear regression analysis between the pathway proportion and genome sizes of the 30 *Asaia* strains included in the study.

### Pyrethroid hydrolase versus species trees cocladogenesis analysis.

The amino acid alignment of the pyrethroid hydrolase gene was retrieved from the output of the previous analyses (see above). The alignment was subjected to ML phylogenetics using RAxML 8 (39) with the LG+G+F model and selected using ProtTest3 (40). The topologies of the pyrethroid hydrolase tree and the species tree obtained by phylogenomic analysis (see above) were then graphically compared using R.

### Data availability.

All the reads related to the new genome sequencing described in this study have been deposited in the European Nucleotide Archive (ENA) with accession number PRJEB43295.
