# Supplementary material for: Phylogenomics Reveals that Asaia Symbionts from Insects Underwent Convergent Genome Reduction, Preserving an Insecticide-Degrading Gene
Source: mBio. 2021 Mar 30;12(2):e00106-21. doi: 10.1128/mBio.00106-21 (PMC8092202; doi:10.1128/mBio.00106-21)
Supplement: TABLE S1 [file mBio.00106-21-st001.pdf]

Table S1: *Asaia* strains information

Origin	Accession_number	Genome_Name	Strain	Host	Host_sex	Location	Origin	Motility
Study	Pending	Asaia_koreicus_male	AkM1	*Aedes koreicus*	male	North Italy	Field	-
Study	Pending	Asaia_koreicus_female	AkF1	*Aedes koreicus*	female	North Italy	Field	+
Study	Pending	Asaia_koreicus_female	AkF3	*Aedes koreicus*	female	North Italy	Field	-
Study	Pending	Asaia_japonicus_male	AjM1	*Aedes japonicus*	male	North Italy	Field	-
Study	Pending	Asaia_japonicus_female	AjF1	*Aedes japonicus*	female	North Italy	Field	+
Study	Pending	Asaia_japonicus_female	AjF2	*Aedes japonicus*	female	North Italy	Field	-
Study	Pending	Asaia_maculipennis_male	AmacM3	*Anopheles maculipennis*	female	North Italy	Field	-
Study	Pending	Asaia_maculipennis_male	AmacM4	*Anopheles maculipennis*	female	North Italy	Field	+
Study	Pending	Asaia_maculipennis_female	AmacF5	*Anopheles maculipennis*	female	North Italy	Field	-
Study	Pending	Asaia_albopictus_male	AaM1	*Aedes albopictus*	unk	North Italy	Field	-
Study	Pending	Asaia_albopictus_male	AaM2	*Aedes albopictus*	male	North Italy	Field	+
Study	Pending	Asaia_albopictus_female	AaF3	*Aedes albopictus*	male	North Italy	Field	+
Study	Pending	Asaia_cer_Guatemala_male	AccGM	*Ceratitis capitata*	male	Guatemala	ISPRA insectarium	+
Study	Pending	Asaia_cer_Guatemala_female	AccGF	*Ceratitis capitata*	female	Guatemala	ISPRA insectarium	+
Study	Pending	Asaia_cer_Ispra_female	AccIF	*Ceratitis capitata*	female	Greece and Italy	ISPRA insectarium	+
Study	Pending	Asaia_cer_La_Reunion male	AccLRM	*Ceratitis capitata*	male	La Réunion	ISPRA insectarium	+
Study	Pending	Asaia_cer_La_Reunion_female	AccLRF	*Ceratitis capitata*	female	La Réunion	ISPRA insectarium	+
PATRIC	1231624.3	Asaia bogorensis NBRC 16594	Abo_NBRC_16594	plant	unk	Indonesia	unk	nd
PATRIC	1236500.5	Asaia astilbis JCM 15831	Aas_JCM_15831	plant	unk	Japan	unk	nd
PATRIC	1236502.5	Asaia prunellae JCM 25354	Apr_JCM_25354	plant	unk	Japan	unk	nd
PATRIC	1236525.5	Asaia platycodi JCM 25414	Apl_JCM_25414	plant	unk	Japan	unk	nd
PATRIC	1382230.3	Asaia platycodi SF2.1	Apl_SF2.1	*Anopheles stephensi*	female	unk	UNICAM insectarium	+
PATRIC	2067395.3	Asaia sp. W19	W19	mosquito	unk	Usa	unk	nd
NCBI	SRX4022631	Asaia_Aedes_albopictus	Aa 5.5	*Aedes albopictus*	female	Central Italy	UNICAM insectarium	+
NCBI	SRX4022630	Asaia_Anopheles_darlingi	Adar	*Anopheles darlingi*	female	Brazil	Field	-
NCBI	SRX4022634	Asaia_Anopheles_funestus	Afun	*Anopheles funestus*	female	Burkina Faso	Field	+
NCBI	SRX4022635	Asaia_Anopheles_gambiae	Agam	*Anopheles gambiae*	female	Burkina Faso	Field	+
NCBI	SRX4022633	Asaia_Anopheles_stephensi	Aaste	*Anopheles stephensi*	female	unk	UNICAM insectarium	+
NCBI	GCA_900465345.1	Asaia_bogorensis_DG-01	GD-01	unk	unk	unk	unk	nd
NCBI	GCA_900465315.1	Asaia_bogorensis_IPC01	IPC-01	unk	unk	unk	unk	nd
NCBI	GCF_000234355.1	Gluconobacter_morbifer	Gmo_G707	unk	unk	unk	unk	nd
PATRIC	634452.3	Acetobacter pasteurianus	IFO 3283-01	unk	unk	unk	unk	nd
PATRIC	1231341.3	Acetobacter orientalis	21F-2	unk	unk	unk	unk	nd
PATRIC	945681.4	Acetobacter pomorum	DM001	unk	unk	unk	unk	nd
PATRIC	1224746.3	Gluconobacter oxydans	H24	unk	unk	unk	unk	nd

PATRIC	1291534.3	Gluconobacter thailandicus	NBRC 3255	unk	unk	unk	unk	nd
PATRIC	1104996.3	Gluconobacter frateurii	NBRC 101659	unk	unk	unk	unk	nd
PATRIC	437.4	Acidomonas methanolica	DSM 5432	unk	unk	unk	unk	nd
PATRIC	1231351.3	Acidomonas methanolica	NBRC 104435	unk	unk	unk	unk	nd
PATRIC	272568.12	Gluconacetobacter diazotrophicus	PAl 5	unk	unk	unk	unk	nd
PATRIC	112140.5	Gluconacetobacter johannae	LMG 21312	unk	unk	unk	unk	nd
PATRIC	1286186.4	Gluconacetobacter aggeris	LMG 27801	unk	unk	unk	unk	nd
