# Supplementary material for: Phylogenomics Reveals that Asaia Symbionts from Insects Underwent Convergent Genome Reduction, Preserving an Insecticide-Degrading Gene
Source: mBio. 2021 Mar 30;12(2):e00106-21. doi: 10.1128/mBio.00106-21 (PMC8092202; doi:10.1128/mBio.00106-21)
Supplement: FIG S1 [file mBio.00106-21-sf001.pdf]

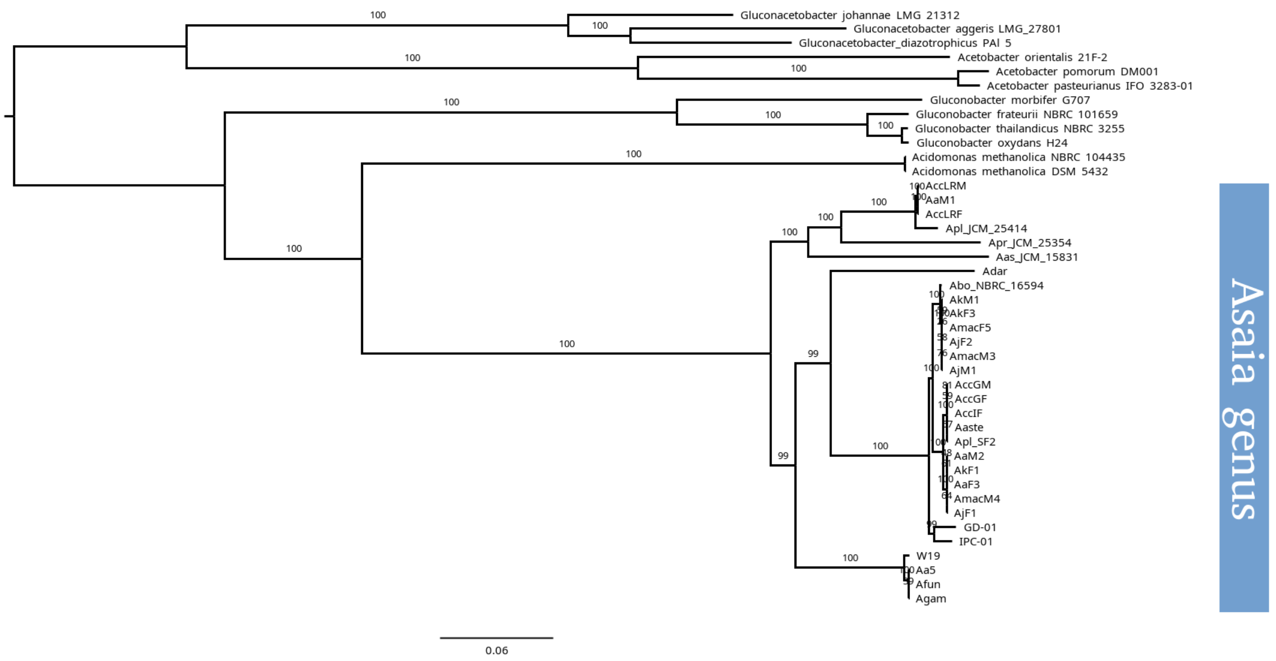

Fig. S1: **Maximum Likelihood phylogenetic tree including multiple outgroup species**. Maximum likelihood phylogenetic tree including the study strains of the genus *Asaia* and 12 strains of four genera belonging to the Acetobacteraceae family. The phylogenetic analysis has been perfomed on a concatenate of 356 core genes. These core genes were selected as follows: present in single copy in all the genomes and without frame shifts or stop codons in the alignment. Bootstrap supporting values are reported on the tree branches.
