# Supplementary material for: Phylogenomics Reveals that Asaia Symbionts from Insects Underwent Convergent Genome Reduction, Preserving an Insecticide-Degrading Gene
Source: mBio. 2021 Mar 30;12(2):e00106-21. doi: 10.1128/mBio.00106-21 (PMC8092202; doi:10.1128/mBio.00106-21)
Supplement: TABLE S2 [file mBio.00106-21-st002.pdf]

Table S2: Pathways linear regression analysis information

COG letter	COG Description	Linear coefficient	P-value	Classification
J	Translation, ribosomal structure and biogenesis	-0,901917832	1,01625E-11	Preserved
I	Lipid transport and metabolism	-0,848446073	3,18876E-09	Preserved
F	Nucleotide transport and metabolism	-0,845795645	3,9953E-09	Preserved
C	Energy production and conversion	-0,815183701	4,12513E-08	Preserved
H	Coenzyme transport and metabolism	-0,778851842	3,99572E-07	Preserved
O	Posttranslational modification, protein turnover, chaperones	-0,70949269	1,13384E-05	Preserved
R	General function prediction only	-0,604620418	0,000401808	Preserved
Q	Secondary metabolites biosynthesis, transport and catabolism	-0,578926619	0,000802969	Preserved
M	Cell wall/membrane/envelope biogenesis	-0,545469386	0,001823972	Preserved
P	Inorganic ion transport and metabolism	-0,522506535	0,00305561	Preserved
E	Amino acid transport and metabolism	-0,48438919	0,006677691	Preserved
T	Signal transduction mechanisms	-0,467280887	0,009227431	Preserved
N	Cell motility	0,383336054	0,036524826	Erosed
L	Replication, recombination and repair	0,647174767	0,000111014	Erosed
K	Transcription	0,677134187	3,96563E-05	Erosed
U	Intracellular trafficking, secretion, and vesicular transport	0,354322184	0,054720558	Unclassified
V	Defense mechanisms	-0,3405282	0,065567818	Unclassified
G	Carbohydrate transport and metabolism	-0,304825409	0,101442222	Unclassified
S	Function unknown	-0,186701426	0,323213587	Unclassified
D	Cell cycle control, cell division, chromosome partitioning	0,013275404	0,944491637	Unclassified
