# Supplementary material for: Phylogenomics Reveals that Asaia Symbionts from Insects Underwent Convergent Genome Reduction, Preserving an Insecticide-Degrading Gene
Source: mBio. 2021 Mar 30;12(2):e00106-21. doi: 10.1128/mBio.00106-21 (PMC8092202; doi:10.1128/mBio.00106-21)
Supplement: TABLE S3 [file mBio.00106-21-st003.pdf]

Table S3: Genome information and statistics

Strain name	Origin	Data type	Accession number	Assembly size	Contig number	Contig mean length	N50	%GC
AkM1	This work	Raw reads	Pending	3249806	45	72217.91	227228	59,70
AkF1	This work	Raw reads	Pending	3395900	31	109545.16	224734	59,68
AkF3	This work	Raw reads	Pending	3214893	24	133953.88	221605	59,91
AjM1	This work	Raw reads	Pending	3213845	23	139732.39	287459	59,92
AjF1	This work	Raw reads	Pending	3395996	29	117103.31	281079	59,68
AjF2	This work	Raw reads	Pending	3212668	20	160633.40	320158	59,92
AmacM3	This work	Raw reads	Pending	3170081	17	186475.35	371960	59,92
AmacM4	This work	Raw reads	Pending	3522271	91	38706.27	187697	59,61
AmacF5	This work	Raw reads	Pending	3214172	28	114791.86	250987	59,92
AaM1	This work	Raw reads	Pending	3534052	27	130890.81	373335	59,11
AaM2	This work	Raw reads	Pending	3396429	31	109562.23	229787	59,68
AaF3	This work	Raw reads	Pending	3427576	30	114252.53	281079	59,69
AccGM	This work	Raw reads	Pending	3526650	34	103725.00	301732	59,76
AccGF	This work	Raw reads	Pending	3524889	32	110152.78	229838	59,76
AccIF	This work	Raw reads	Pending	3524733	32	110147.91	262207	59,76
AccLRM	This work	Raw reads	Pending	3524501	19	185500.05	373265	59,11
AccLRF	This work	Raw reads	Pending	3526893	31	113770.74	279785	59,11
Abo_NBRC_16594	PATRIC	Assembly	1231624.3	3198265	1	3198265.00	3198265	59,81
Aas_JCM_15831	PATRIC	Assembly	1236500.5	3142477	43	73080.86	146992	58,03
Apr_JCM_25354	PATRIC	Assembly	1236502.5	3180419	35	90869.11	192977	55,77
Apl_JCM_25414	PATRIC	Assembly	1236525.5	3147101	30	104903.37	117874	59,30
Apl_SF2.1	PATRIC	Assembly	1382230.3	3420650	27	126690.74	337172	59,89
W19	PATRIC	Assembly	2067395.3	3903531	187	20874.50	140985	60,35
Aa 5.5	NCBI	Raw reads	SRX4022631	3789637	46	82383.41	393674	60,36
Adar	NCBI	Raw reads	SRX4022630	3082998	11	280272.55	512449	61,10
Afun	NCBI	Raw reads	SRX4022634	3602720	65	55426.46	139564	60,46
Agam	NCBI	Raw reads	SRX4022635	3687255	68	54224.34	504831	60,43
Aaste	NCBI	Raw reads	SRX4022633	3525005	28	125893.04	397297	59,76
GD-01	NCBI	Assembly	GCA_900465345.1	3375843	42	80377.21	156758	59,77
IPC-01	NCBI	Assembly	GCA_900465315.1	3166761	19	166671.63	284403	59,69
Gmo_G707	NCBI	Assembly	GCF_000234355.1	2887061	19	151950.58	422539	59,04
